# Recent Advances in Novel Compositions for Electrochemical Applications

**DOI:** 10.3390/ijms242015388

**Published:** 2023-10-20

**Authors:** Almaz Zagidullin, Mikhail Khrizanforov

**Affiliations:** Arbuzov Institute of Organic and Physical Chemistry, FRC Kazan Scientific Center, Russian Academy of Sciences, Arbuzov Str. 8, 420088 Kazan, Russia

## 1. Introduction

In recent years, there has been a significant rise in innovative developments in the field of electrochemical composites [1,2,3,4]. These developments have focused on both organic [5,6,7] and inorganic compounds [8,9,10,11,12], including coordination polymers [13,14,15,16]. The importance of these novel compositions cannot be overstated, as they are crucial for the development of a range of molecular materials and devices [17,18]. These advancements have led to the creation of electrocatalysts [19,20], electrochemical sensors [21,22], and fuel cells [23], among other things. The state-of-the-art advancements in the field have made it possible to create more efficient materials and devices, which have the potential to revolutionize various industries. The potential applications of these electrochemical compositions are vast, and they are expected to play a significant role in shaping the nearest technologies. As such, the research and development of novel compositions for electrochemical applications are of significant importance.

This collection of articles presents extensive research and discoveries that leverage various morphologies of electrochemical compositions, emphasizing their multifunctional properties, such as advanced redox properties [24,25,26,27,28,29,30,31]. The published articles range from the drug design and synthesis of hybrids of sterically hindered phenols and diaryl ureas [31] to sophisticated studies on the enhancement of arc erosion resistance in AgCuO electrical contact materials [30]. Other notable topics include in-depth research on chemical and electrochemical reductions of monoiminoacenaphthenes [29], investigations on conductive mediators based on ferrocene functionalized phosphonium ionic liquids [26], and critical assessments of salt stress based on innovative electrochemical sensor detection methodologies [24].

## 2. An Overview of Published Articles

In the study of salt stress on *Arabidopsis*, X. Du and co-workers utilized an electrochemical sensor to detect hydrogen peroxide released by plant cells [24]. The sensor was modified with a nanocomposite made of MWCNT-Ti_3_C_2_T_x_-Pd, which enhanced the signal of the electrode. The nanocomposite was characterized using a transmission electron microscope, which revealed that the MWCNTs were fibrous and could reach the micron level, while the Ti_3_C_2_T_x_ presented multilayer flake, expanding the electroactive area and providing more attachment sites for Pd nanoparticles. To test the stability of the electrochemical sensor, the modified electrodes were placed in a 5 mM H_2_O_2_ solution and measured every two days. The electrodes still reacted well to H_2_O_2_ on the sixth day, indicating a good stability and laying a foundation for the long-term determination of H_2_O_2_ released from leaves. Overall, the use of an electrochemical sensor provided a reliable and sensitive method for detecting hydrogen peroxide in plant cells, which can be used to assess the impact of salt stress on metabolic processes.

The research work of M. Kadirov’s group investigates the use of sodium pectate–nickel complexes as catalysts in fuel cells for hydrogen oxidation and oxygen reduction [25]. The study aimed to determine the catalytic activity of these complexes and their potential as a renewable energy source. The researchers utilized electrochemical techniques, including a rotating disk electrode and the Koutecky–Levich equation, to investigate the kinetics of the oxygen electroreduction reaction. The results showed that the sodium pectate–nickel complexes exhibited promising catalytic activity for oxygen reduction. The study provides valuable insights into the use of these complexes as catalysts in fuel cells, which could have significant implications for the field of renewable energy. The research also highlights the importance of developing sustainable and efficient energy sources to address the growing demand for energy and reduce the impact of fossil fuels on the environment.

In the work of V. Ermolaev et al., the synthesis and extensive characterization of ferrocene functionalized phosphonium ionic liquids were described [26]. The team endeavors to shed light on the plausible applications of such salts, particularly focusing on their role as conductive mediators within the scope of oxidation reactions. The research employed the meticulous technique of cyclic voltammetry to examine the electrochemical attributes inherent to these specialized salts. The investigation revealed that the metamorphosis of C=O groups into CH_2_ fragments exerted a considerable impact on the salts’ electrochemical dynamics. Intriguingly, variations in the length of the alkyl chain did not instigate substantial shifts in potential, maintaining a relative consistency in their electrochemical characteristics. Beyond the immediate findings, this piece of research extends its relevance to broader scientific domains, encompassing both organic chemistry and materials science. The elucidated insights and unveiled potentials of ferrocene-containing phosphonium salts as conductive mediators set the stage for future innovations and applications in these scientific fields. The investigation opens up avenues for further studies into the properties and applications of ferrocene-functionalized salts, probing deeper into their potential roles and impacts in varied chemical reactions and material compositions.

Y. Budnikova et al. brought forth exploring the application of non-noble-metal mono- and bimetallic composites as efficient catalysts in the electrochemical processes involving phosphine oxide and acetylene C-H/P-H coupling under relatively mild conditions [27]. This research aims to offer groundbreaking insights into the world of electrochemical phosphorylation, especially into compounds like phenylacetylene, using innovative catalytic nanoparticles derived from non-noble metals, paving the way for a new dimension in electrocatalysis. The study meticulously delves into the redox properties of these specially designed nanoparticles. Employing cyclic voltammetry, the research analyzes these properties on glassy carbon electrodes that underwent modification. This technique unveils the intricate dynamics and inherent properties of the nanoparticles, allowing for a more nuanced understanding of their electrochemical behavior and potential applications. Beyond the specific findings, this research introduces a synthetic protocol that bears significant potential for applicability in other atom-economical reactions occurring at room temperature. The work represents a significant stride in the field of electrochemistry, providing a richer, more in-depth exploration of the properties and potentials of non-noble-metal-based nanoparticles as electrocatalysts.

The work of Yakhvarov group unveils a pioneering approach to constructing functionalized phosphorene nanosheets, a groundbreaking achievement in the world of nanomaterials [28]. This ingenious process involves an intricate combination of in situ electrochemical exfoliation and methylation applied to black phosphorus, ultimately culminating in the creation of nanosheets imbued with remarkable functionalities. Through a carefully controlled electrochemical dance, the black phosphorus undergoes a transformation, giving rise to exfoliated BP nanosheets. However, the transformation is not complete without the subsequent electrochemical methylation step. In a separate electrochemical cell, the freshly exfoliated BP nanosheets are subjected to the touch of a methylating agent CH_3_I. This chemical interaction confers a suite of novel properties upon the nanosheets, elevating them from a simple to a sophisticated functional material. Perhaps one of the most promising aspects of this groundbreaking method lies in its scalability. The process exhibits a remarkable potential for industrial production, hinting at a future where functionalized phosphorene nanosheets could become readily accessible for a wide range of applications.

The research of V. Khrizanforova et al. delves deep into the fascinating realm of electrochemistry, specifically focusing on the electrochemical reduction of monoiminoacenaphthenes (MIANs) [29]. Employing a diverse array of techniques, including cyclic voltammetry (CV) and differential pulse voltammetry (DPV), this study meticulously examines the complex electrochemical landscape of MIANs. One of the primary objectives of this investigation was to unravel the intricate redox properties exhibited by MIANs and shed light on their electrochemical behavior. MIANs showcase three distinctive reduction peaks within the cathode potential range. The notable difference between the anodic and cathodic potentials points towards a high degree of electrochemical reversibility in the electron transfer processes occurring within the ligand. Of particular interest is the first oxidation peak, which corresponds to the formation of the cation radical. Interestingly, this particular process is found to be irreversible, adding an intriguing layer of complexity to the electrochemical behavior of MIANs. These findings, elucidated through careful experimentation and analysis, offer valuable insights into the electrochemical reduction of MIANs. Beyond the realm of fundamental research, the knowledge gleaned from this study holds immense promise for a myriad of practical applications across various scientific fields.

H. Wang and colleagues embarked on a pioneering exploration aimed at enhancing the arc erosion resistance of AgCuO electrical contact materials through the strategic incorporation of rare earth elements [30]. This comprehensive investigation leveraged a synergistic approach, combining first-principle calculations with a suite of experimental techniques. Their objective was to delve into the intricacies of the stability, electrical, and physical properties of both undoped CuO and CuO enriched with rare earth elements. To achieve this, the research team conducted arc erosion tests on these specially engineered electrical contacts. They meticulously analyzed the consequential alterations in their mass and morphology using a battery of sophisticated tools, including electronic analytical balances, cutting-edge three-dimensional profilers, high-resolution scanning electron microscopy, and precise energy-dispersive X-ray spectroscopy. By introducing rare earth element doping, researchers have uncovered novel strategies for enhancing the performance and durability of electrical contact materials. These insights hold significant implications for a wide array of applications, spanning from the design of more robust electrical components to the advancement of electrical engineering as a whole.

I. Alabugin’s research group in electrochemistry challenges the classical understanding of antioxidants and their properties [31]. By employing cyclic voltammetry with a semi-differential method, they unveiled new avenues in the quest for compounds that exhibit both antioxidant and anti-tumor properties. This innovative approach enables a comprehensive assessment of the reducing capacity and electrochemical behavior of phenolic antioxidants. It also facilitates the identification of multiple redox-active groups within sterically hindered phenols and diaryl urea hybrids. The insights garnered from these electrochemical measurements shed light on the intricate relationship between antioxidant and pro-oxidant activities and oxidation potential. This includes the ability to donate electrons and intercept free radicals, which are key factors in their effectiveness. This research underscores the potential of electrochemistry as a powerful tool for advancing our understanding of antioxidants and their dual roles in mitigating oxidative stress and promoting health. Moreover, these findings have far-reaching implications beyond fundamental science. The ability to design compounds with tailored antioxidant properties, informed by electrochemical insights, holds promise for the creation of innovative therapies and products that address pressing challenges in drug design and healthcare.

## 3. Conclusions

The articles in this collection showcase the innovative research and development of novel compositions for the creation of electrocatalysts, electrochemical sensors, and fuel cells, as well as in-depth studies on electrochemical reductions, investigations on conductive mediators, and the relationship between antioxidant activities and oxidation potential for drug design. This spectrum of research articles not only offers diverse insights into the field of electrochemical applications, but also serves as an impetus for further interdisciplinary research and discoveries and collaborations across various fields. Overall, this collection of articles provides valuable insights into the exciting potential of electrochemical compositions and their applications in shaping the future of technologies, making it an essential resource for researchers, scientists, and industry professionals.
